# Three Complete Mitochondrial Genomes of Erotylidae (Coleoptera: Cucujoidea) with Higher Phylogenetic Analysis

**DOI:** 10.3390/insects12060524

**Published:** 2021-06-05

**Authors:** Jing Liu, Yuyu Wang, Ruyue Zhang, Chengmin Shi, Weicheng Lu, Jing Li, Ming Bai

**Affiliations:** 1 College of Plant Protection, Hebei Agricultural University, Baoding 071001, China; liujing15231147432@126.com (J.L.); zhbwyy@hebau.edu.cn (Y.W.); zry47534@163.com (R.Z.); shichengmin@hebau.edu.cn (C.S.); 15632242386@163.com (W.L.); 2 Key Laboratory of Zoological Systematics and Evolution, Institute of Zoology, Chinese Academy of Sciences, Beijing 100101, China

**Keywords:** mitochondrial genome, Erotylidae, phylogenetic relationships

## Abstract

**Simple Summary:**

Erotylid beetles are phytophagous and mycophagous. Phylogenetic studies on this family were mainly based on morphological characters or several gene fragments. Research on the mitochondrial genome of Erotylidae is rare. Therefore, we sequenced and analyzed three complete mt genomes of Erotylinae with a comparative mt genomic analysis of Erotylinae and Languriinae for the first time to reveal mitochondrial genome characterizations and reconstruct phylogenetic relationships of this group. The comparative analyses showed the mt genome characterizations of Erotylinae are similar to Languriinae. These results provided a comprehensive framework and worthy information for the future research of this family.

**Abstract:**

The family Erotylidae belongs to the superfamily Cucujoidea, which are phytophagous and mycophagous. So far, only two representative complete mitochondrial (mt) genomes of Erotylidae have been sequenced. Mitochondrial genomes of *Tritoma metasobrina*, *Neotriplax arisana*, and *Episcapha opaca*, which all belong to the subfamily Erotylinae, were sequenced using next-generation sequencing technology to better understand the diversity of mt genomes of Erotylidae. A comparative mt genomic analysis was conducted on the three sequenced representatives of Erotylinae and Languriinae sp. (Languriinae). The size of the complete mt genome of the 4 species ranged from 15,581 bp to 16,502 bp in length, including 37 genes (13 protein-coding genes, 22 transfer RNAs, and 2 ribosomal RNAs) and the control region. The arrangements of their mt genomes are highly consistent with other Coleoptera species. The start codons of two PCGs (*ND1* and *ND5*) and the stop codons of one PCG (*ATP8*) were illustrated differences between Languriinae sp. and the other three species. All tRNAs of these 4 species exhibited cloverleaf secondary structures except that the dihydorouridine (DHU) arm of *tRNA^Ser(AGN)^* was absent. The phylogenetic analyses using both Bayesian inference (BI) and maximum likelihood (ML) methods all supported that Erotylidae as monophyletic. Erotylinae was monophyletic being the sister group to Xenocelinae. Languriinae was closely related to ‘Erotylinae-Xenocelinae’. Our results recovered Languriinae nested within Erotylidae.

## 1. Introduction

The diverse family Erotylidae belongs to the superfamily Cucujoidea (Coleoptera: Polyphaga), which includes about 260 genera and 3500 species in the world [1]. The larvae and adults of Erotylidae have different feeding habits [2]. The highest numbers of species are contained in the basidiomycete fungus-feeding Erotylinae and the mainly phytophagous-feeding Languriinae, whereas other subfamilies include fewer species with mixed diets [2]. The monophyly and composition of Erotylidae have never been explicitly determined since Latreille established this family [3]. The monophyly of Erotylidae was supported by Leschen and Lawrence based on morphological analysis of Cucujoidea [4,5,6]. In Leschen’s study, the sister group with Erotylidae was not clear [4]. The monophyly of the family Erotylidae was also put forward by Hunt and Mcelrath according to the molecular phylogenetic study [7,8] and the results showed Erotylidae was closely related to Protocucujoidae, Helotidae, and Monotomidae. But the paraphyly of Erotylidae has been recovered by a molecular phylogeny based on several nuclear and mitochondrial genes [9]. The paraphyletic or polyphyletic Erotylidae was also proposed by the morphological characters in Cucujoidea [10].

The relationships within Erotylidae are still controversial, especially between Erotylinae and Languriinae. Languriidae should be merged into Erotylidae as indicated by Crotch as well as Wegrzynowicz and Leschen [11,12,13]. This conclusion was also supported by Robertson, who analyzed the *18S rRNA* and *28S rRNA* gene sequences of 61 species [14]. But there has also been some research that showed Languriidae and Erotylidae to be separate families [15,16,17,18,19]. Arrow showed that there were significant morphological differences between Erotylidae and Languriidae, such as the procoxal cavities open and the larval spiracles are divided into two pairs in Languriidae, whereas in Erotylidae the procoxal cavities are closed and the larval spiracles are of a simple annular shape [15]. This result was also supported by Crowson, Sen, and Leschen [16,17,18]. Kai indicated that Languriidae should be regarded as a separated family based on their feeding characteristics [19]. So, the phylogenetic position of Languriinae and the internal phylogenetic relationships of Erotylidae are still unclear.

Phylogenetic investigations of Erotylidae have remained at the level of morphology and mitochondrial (mt) markers for a long time. In recent years, mt genome has been widely used in the analysis of species heredity and molecular evolution due to their advantages such as small molecular weight, ease to operate, fast mutation speed, and maternal inheritance [20,21,22]. The complete mt genome can provide more informative genetic information as well as a suite of genomic-level characters, such as RNA secondary structures and gene arrangements in comparison with the individual mt genes. [23,24].

Currently, the study of mt genomes from Erotylidae is still scarce. A total of 10 mt genomes label as Erotylidae were available in GenBank (accession date, 9 January 2021). Of them, one mt genome (accession No. KT696227.1) was likely mislabeled, because manifested high sequence identity (99% for *COX1* gene) with *Thamiaraea americana*. Another 8 mt genomes for 6 unidentified species of Erotylidae sp., *Loberonotha olivascens,* and *Tritoma bipustulata*, are highly incomplete. Here, the mt genomes of three species, *Tritoma metasobrina* Chûjô, 1941, *Neotriplax arisana* Miwa, 1929 and *Episcapha opaca* Heller, 1920 were sequenced using next-generation sequencing to better understand the diversity of mt genomes and the phylogenetic of Erotylidae [25,26,27]. A comparative mt genomic analysis of two subfamilies in Erotylidae was conducted including *T. metasobrina*, *E. opaca*, *N. arisana* (Erotylinae), and Languriinae sp. (Languriinae). The results will lay a foundation for the study of Erotylidae mitogenomes. The phylogenetic relationships were reconstructed to demonstrate the phylogenetic position of Erotylidae as well as to explore the phylogeny of Cucujoidea. The results will lay a foundation for the study of Erotylidae as well as Cucujoidea.

## 2. Materials and Methods

### 2.1. Sampling and Genomic DNA Extraction

Specimens of *T. metasobrina* (September 2019, 113°46′13″ E, 36°50′47″ N), *E. opaca* (April 2020, 102°37′30″ E, 24°56′17″ N), and *N. arisana* (September 2019, 101°56′26″ E, 35°16′36″ N) were collected in Yunnan, Qinghai and Hebei, respectively. Specimens were preserved in absolute ethanol at −20 °C for storage at the Hebei Agricultural University (HEBAU). Genomic DNA was extracted by DNeasy Blood & Tissue kit (QIAGEN, Hilden, Germany) (Table S1).

### 2.2. Genome Sequencing and Analyses

DNA quality and quantification were determined by the ND-2000 (NanoDrop Technologies). Only high-quality (OD260/280 = 1.8–2.0, OD260/230 ≥ 2.0) and sufficient DNA sample was used in subsequent experiments.

Sequencing libraries (Illumina NovaSeq) were prepared using genomic DNA with an average insert size of 400 bp, and were sequenced on the Illumina Hiseq platform with 150 bp paired-end reads at Majorbio (Shanghai, China). According to the principle of second-generation sequencing data, the original offline data (Raw Data) was subjected to fastp [28] quality control filtering to obtain Clean Data, and the analysis of Clean Data was performed according to GATK Best Practice for mutation detection. MitoZ [29] was used to conduct assembly and annotation, then checked by manual proofreading according to its relative species. TRNA scan-SE Search Server v1.21 [30] was used to identify the tRNA genes and then manually proofread. The secondary structures of two rRNAs (*rrnS*, *rrnL*) were predicted by RNA Structure (http://rna.urmc.rochester.edu/RNAstructureWeb/) (accessed on 25 December 2020). [31]. All PCGs were annotated by alignment with homologous genes from *T. metasobrina*, *N. arisana*, and *E. opaca* using Geneious 8.0.5 software (Biomatters, Auckland, New Zealand) [32]. MEGA 7.0 was used to calculate the A + T content and relative synonymous codon usage (RSCU) for PCG analysis [33]. The bias of base usage was measured by AT-skew and was calculated to AT-skew = (A − T)/ (A + T) [34]. The numbers of synonymous substitutions (Ks) and non-synonymous substitutions (Ka), and the ratios of Ka/Ks for each PCG were also measured in the software KaKs_Calculator 2.0. [35] (Table S1). MEGA 7.0 [33] was used to analyze the genetic distances based on Kimura-2-parameter among the 4 mt genomes were analyzed by. Genome organization and base composition, PCGs, RSCU, tRNAs, rRNAs, CR, intergenic spacer, and overlapping regions of the mt genomes were compared between Erotylidae species. The newly sequenced mitogenome sequences of *T. metasobrina*, *E. opaca*, and *N. arisana* were submitted to GenBank with the accession numbers MZ014622, MZ014623, and MZ014624, respectively.

### 2.3. Phylogenetic Analyses

In this study, the phylogenetic analyses were conducted based on 17 mt genomes from GenBank (Available online: http://www.ncbi.nlm.nih.gov) (accessed on 9 January 2021). including three newly sequenced (Table 1). Most of the mt genomes chosen were complete. The ingroup taxa included 15 species from Cucujoidea representing 7 families and the outgroup taxa included Curculionidae and Chrysomelidae for their close relationships with Cucujoidea [36,37].

Clustal_X [38] was used to conduct DNA alignment from the amino acid alignment of PCGs. MEGA 7.0 was used to connect all alignment sequences excluding the stop codon. Bayesian inferences were conducted with PhyloBayes 3 [39] on DNA sequence alignment with the CAT-GTR model, and on the amino acid alignment with the CAT-Poisson model [40,41,42], respectively. Two Markov chain Monte Carlo (MCMC) chains were employed. The maximum likelihood analysis was conducted with IQ-Tree in Phylosuite [43,44] on DNA sequence alignment with the GTR + F + I + G4 model selected by ModelFinder [45]. Branch supports were evaluated through the ultra-fast bootstrapping method [46] with 1000 replicates (Table S1). The inferred phylogenetic trees were viewed and illustrated using FigTree [47].

## 3. Results and Discussion

### 3.1. General Features of Erotylidae mt Genomes

**Figure 1:**
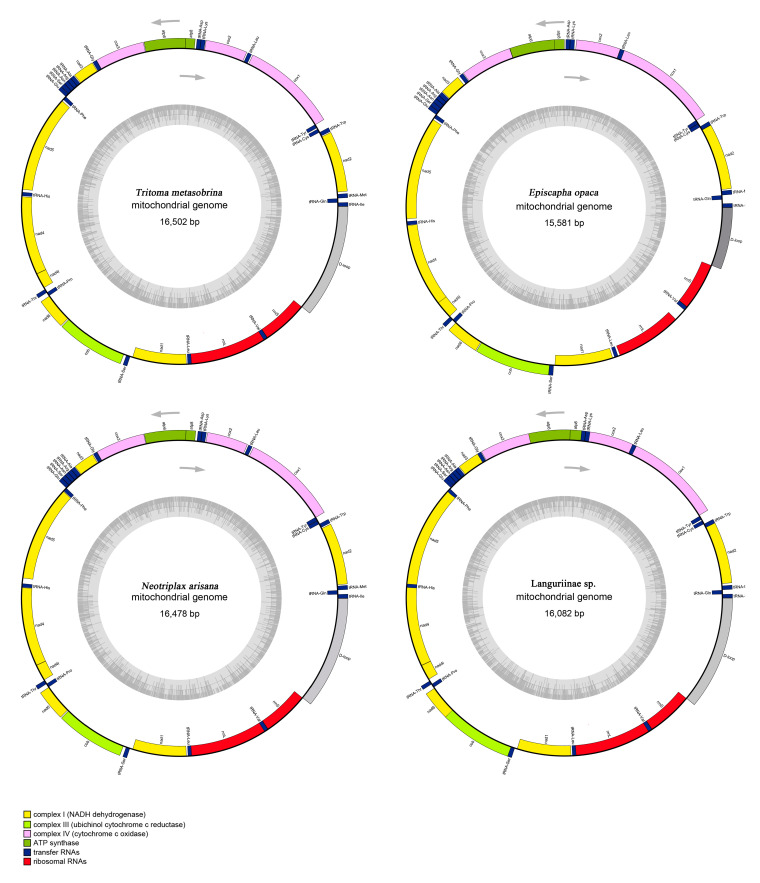
Mitochondrial map of *T. metasobrina*, *E. opaca*, *N. Arisana,* and Languriinae sp. The tRNAs, rRNAs, and PCGs are denoted by the color blocks. Genes outside the map are transcribed clockwise, whereas those inside are transcribed counterclockwise.

Including the newly sequenced mt genomes of *T. metasobrina, E. opaca, and N. arisana*, a total of four mt genomes representing three tribes and two subfamilies of Erotylidae were used in our comparative analyses. The mt genomes of four specimens were typical circular double-strand molecules, ranging from 15,581 bp to 16,502 bp in length. The mt genomes included 13 Protein-coding genes (PCGs), 22 transfer RNAs (tRNAs) and 2 ribosomal RNAs (rRNAs), and the control region (CR) (Figure 1). Twenty-three genes were encoded at the majority strand (J), while the remaining 14 genes were oriented at the minority strand (N). The 4 species displayed a 929–1785 bp CR, identified by the position between the *rrnS* and *tRNA^Ile^.* The overlap nucleotides were located between *tRNA^Tyr^*/*COX1* of *T. metasobrina* (8 bp), *tRNA^Trp^*/*tRNA^Cys^* of *E. opaca* (8 bp), *ATP8*/*ATP6* of *N. arisana* (4 bp) and *ATP8*/*ATP6* and *ND4/ND4L* of Languriinae sp. (7 bp). In addition to the CR, the largest non-coding region of these 4 species was located between *tRNA^Ile^* and *tRNA^Gln^* in *T. metasobrina* with a length of 145 bp (Table 2).

The mt genomes of Erotylidae had a significant bias towards A and T, with the nucleotide composition of A and T in total ranging from 74.80% in *T. metasobrina* to 78.70% in *E. opaca* [48]. The content of A + T in the PCGs, tRNAs, rRNAs and CR were ranging from 72.90% to 77.79%, 77.48% to 79.20%, 80.20% to 80.4% and 75.50% to 81.59%. AT-skews and GC-skews were used to measure the strand bias of nucleotide composition of metazoans mt genomes [49]. The AT-skew was −0.01 to 0.04 and the GC-skew was −0.29 to −0.20 (Table 3).

### 3.2. Protein-Coding Genes

Most PCGs started with ATN, except for several genes that started with GTG or TTG. The PCGs stopped with TAA/TAG or truncated termination codons with T/TA-tRNA [50]. The start codons of two PCGs (*ND1* and *ND5*) and stop codons of one PCG (*ATP8*) were illustrated differences between Languriinae sp. and the other three species (Table 2).

**Figure 2:**
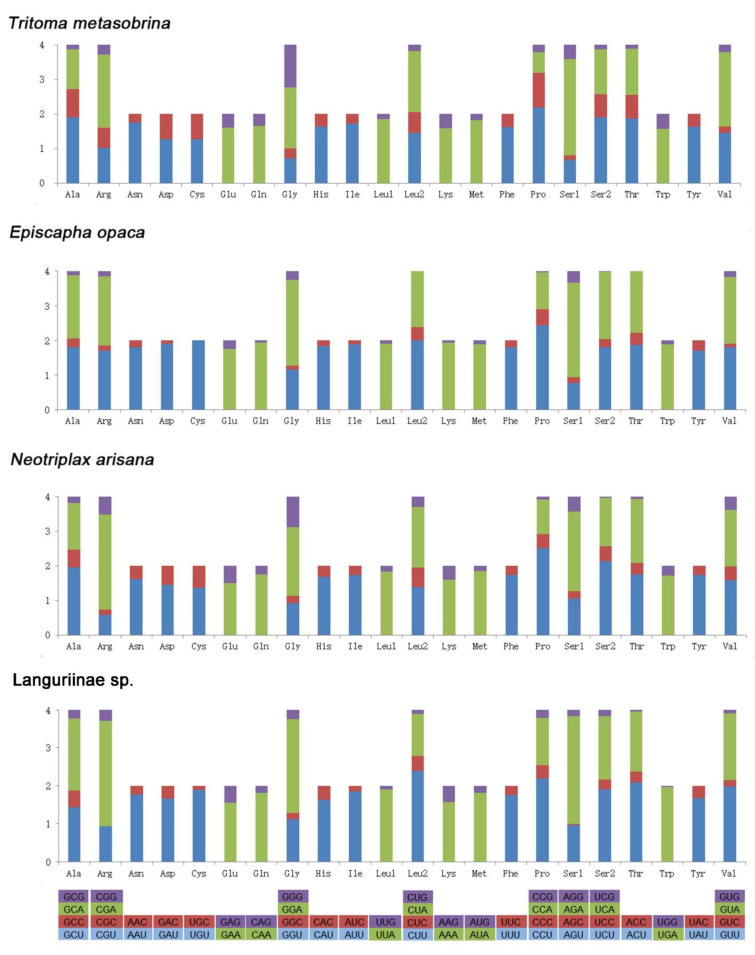
Relative synonymous codon usage (RSCU) of 4 species. Codon families are provided on the x-axis.

The number of each codon, the amino acid(aa) compositions of PCGs, and relative synonymous codon usage (RSCU) values in four species were given in Figure 2. A total of 3641–3691 codons were used excluding termination codons. The frequency of A and U in the third site of four species was much higher than C and G. The four most frequently used codons were CGA, AGA, CCU, and GGA. The highest value of RSCU was 2.84 of AGA in Languriinae sp. The lowest value of RSCU was 0 of UGC and CUG in *E. opaca*. Leucine, Isoleucine, Phenylalanine, and Methionine were the most frequent coding amino acids (aa). The most frequently used codons were TTA, ATT, TTT, and ATA indicating the preference of nucleotide composition A/T.

**Figure 3:**
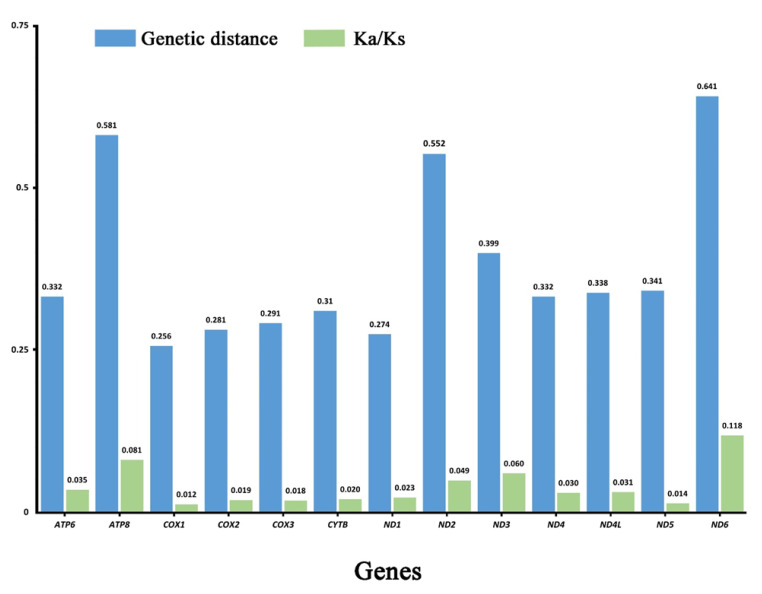
Genetic distance (on average) and non-synonymous (Ka) to synonymous (Ks) substitution rates of 13 PCGs among these 4 species.

Analysis of pairwise genetic distance showed similar results with *ND6* (0.641), *ATP8* (0.581), and *ND2* (0.552) evolving relatively faster, while *COX1* (0.256) and *ND1* (0.274) were slower (Figure 3). Average non-synonymous (Ka)/synonymous (Ks) substitution rate ratios can be used to estimate the evolutionary rate of mitogenome PCGs [51]. We calculated Ka/Ks ratios for each PCG of 4 species in this study, which was shown in Figure 3. Ratios ranged from 0.012 for *COX1* to 0.118 for *ND6*, which indicated that all PCGs were under purifying selection [52]. Our results showed *ND6* and *ATP8* exhibited relaxed purifying selection, while *COX1* was under the strongest purifying selection. Analyses of nucleotide diversity, genetic distance, and evolutionary rate are useful for designing specific markers among different groups. Like other Coleoptera [53], our comprehensive analysis showed that *COX1* had the lowest evolution rate and evolves under comparative relaxed purifying selection, two genes (*ND6* and *ATP8*) exhibited a faster evolution rate and diversity than other PCGs.

### 3.3. Transfer RNAs, Ribosomal RNAs, and Control Region

**Figure 4:**
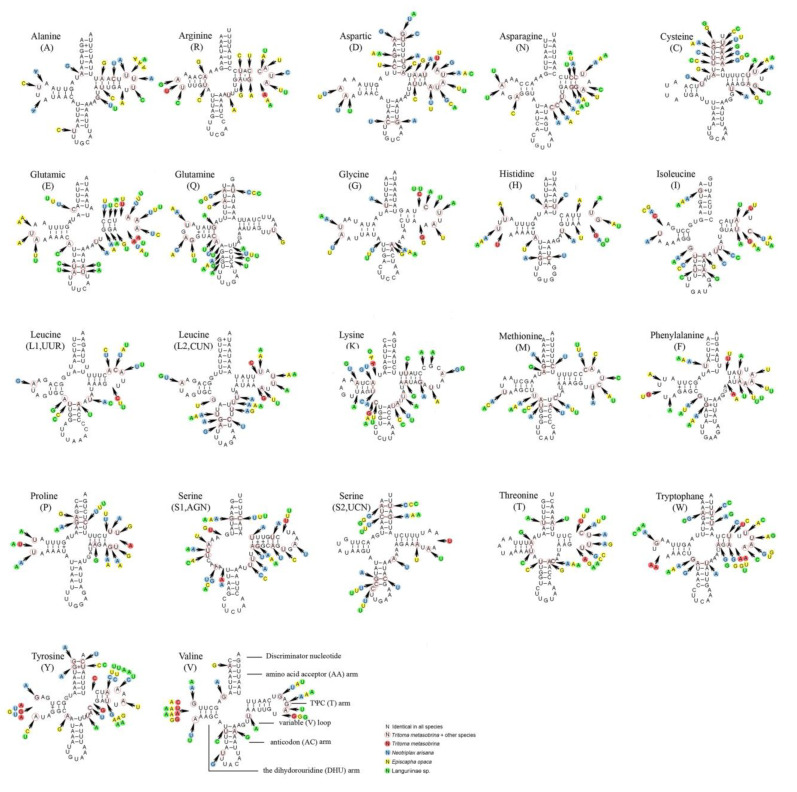
Inferred secondary structures of 22 tRNAs of 4 species. The tRNAs are labeled with the abbreviations of their corresponding amino acids. Dash (–) indicates Watson-Crick bonds and symbol (+) indicates GU bonds.

The predicted secondary structures of tRNAs were shown in Figure 4. The 22 tRNAs of four species were typical and included all 20 types of amino acids (aa). Almost all tRNAs could be folded into cloverleaf structures except *tRNA^Ser(AGN)^* whose DHU arm simply formed a loop. In the tRNAs secondary structure of the 4 species, the anticodons were consistent. The anticodon of *tRNA^Ser (AGN)^* was UCU, which was not a common GCU. UCU was used as the anticodon for the polyphagia group, which was the common form of the metazoans [48]. The aminoacyl (AA) stem length was conservatively 7 bp. Anticodon (AC) arm length was 5 bp except for *tRNA^Lys^*, *tRNA^Met^,* and *tRNA^Leu(UUR)^*. Almost all tRNAs had the same length of the anticodon (AC) loop (7 nucleotides) except *tRNA^Leu(UUR)^* (9 nucleotides). The length of the TψC arm varied from 4 to 6 bp while the TψC loop from 3 to 11 nucleotides. The dihydrouridine (DHU) stem varied from 3 to 4 bp except *for tRNA^Ser(AGN)^*. 11–13 pairs of G-U which form weak attraction and constitute bonds situated at the T arm, the AA arm, the AC arm, and the DHU arm, respectively.

The *rrnL* was located in *tRNA^Leu (CUN)^* and *tRNA^Val^*, and its length ranged from 1052 to 1379 bp. The longest *rrnL* was shown in Languriinae sp. The shortest *rrnL* was shown in *E. opaca*. The *rrnS* was located in *tRNA^Val^* and CR in the four mt genomes of Erotylidae with the length ranging from 759 to 777 bp. The shortest *rrnS* was shown in *E. opaca*. The longest *rrnS* was shown in *N. arisana*. The AT content of *rrnL* and *rrnS* ranged from 80.70% to 81.70% and 77.90% to 81.03%, respectively, which showed a high AT bias. The highest AT content of *rrnL* and *rrnS* was found in *T. metasobrina* and *E. opaca* (Table 3).

The largest noncoding region located between *rrnS* and *tRNA^Ile^* was CR, which is the place that controls replication and transcription [54,55]. The length of CRs were determined and ranged from 929 bp in *E. opaca* to 1785 bp in *T. metasobrina* (Figure 1). This region contained the highest proportion of A and T, ranging from 75.5% to 81.59%. The AT-skew was −0.02 to 0.17 and the GC-skew was −0.66 to −0.27, which indicated that A and T are more numerous than C and G (Table 3).

### 3.4. Phylogenetic Analyses

**Figure 5:**
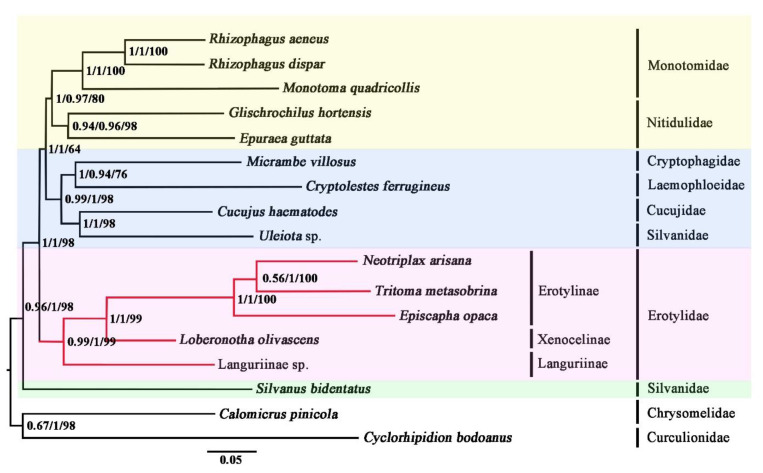
Phylogenetic relationships among Cucujoidea. Shown here is the phylogeny inferred from the AA dataset using PhyloBayes with the CAT-Poisson model. Analyses of the PCG123R dataset using both IQ-Tree with the GTR + F + I + G4 model and PhyloBayes with the CAT-GTR model obtained the same topology as shown here. The first values at each node represent the posterior probabilities from PhyloBayes analysis of the AA dataset with the CAT-Poisson, the second represents the posterior probabilities from PhyloBayes analysis of the PCG123R dataset with the CAT-GTR model, and the third values represent bootstrap supports from IQ-Tree analysis of the PCG123R dataset with the GTR + F + I + G4 model.

Phylogenetic analyses were performed both on the nucleotide dataset (PCG123R) and the amino acid dataset (AA). The PCG123R dataset includes 12,592 sites for 13 PCGs and two rRNA genes. The AA dataset is composed of 3592 amino acids translated from the PCGs. The results of phylogenetic analyses were shown in Figure 5. Irrespective of the methods used, analyses on the PCG123R dataset and the AA dataset reached the same topology (Figure 5). Most nodes were highly supported.

Erotylidae was recovered as monophyletic in all analyses. This result was also supported based on morphological characters [4,5,6] and molecular evidence [7,8]. The sister-group relationship between Erotylidae and “Cryptophagidae-Laemophloeidae-Cucujidae-Silvanidae’-‘Monotomidae-Nitidulidae’” was highly supported and together being the sister group to *Silvanus bidentatus* (Silvanidae) in all analyses. The result was also supported by analyzing nuclear protein-coding genes in Coleoptera [56]. Within the family Erotylidae, three species of Erotylinae were as a monophyletic group, sister to Xenoceninae. The sister relationship between Languriinae and other sampled subfamilies (Erotylinae and Xenocelinae) was recovered by analyses of both BI and ML. The result showed that Languriinae should belong to Erotylidae, which supported the earlier results from Crotch, Leschen, Wegrzynowicz, and Robertson [11,12,13,14].

## 4. Conclusions

The mt genomes sequences of *T. metasobrina*, *E. opaca*, *N. arisana*, and Languriinae sp. were obtained by next-generation sequencing. Their mt genomes had a total length of 16,502 bp in *T. metasobrina*, 15,581 bp in *E. opaca*, 16,478 bp in *N. arisana,* and 16,082 bp in Languriinae sp., respectively. Each of the mt genomes was composed of 13 PCGs, 2 rRNAs, 22 tRNAs, and a control region. In general, mt genomes for members of Erotylidae were similar in genome size and gene order to other species of Cucujoidea. The base composition was biased towards A and T as was commonly found in other insects [48]. However, the content of A + T is the highest in *rrnL* among four species of Erotylidae were have compared here. Most PCGs were initiated with the typical ATT/ATG codon and terminated with TAA/TAG or T/TA-tRNA. However, the start codon for *ND1* of all three species of Erotylinae, *T. metasobrina*, *E. opaca,* and *N. arisana* is TTG, while that for species of Languriinae is ATT. The stop codons for *ATP8* are TAG in Languriinae species but are TAA in Erotylinae species. The start codons of *ND5* were GTG in Languriinae sp., not typical ATN condon [47]. The phylogenetic reconstructions with BI and ML methods all supported the monophyly of Erotylidae and Erotylinae. This representative of Languriinae fitted well to the clade Erotylidae as shown in previous research. This subfamily was recovered being the sister group to ‘Erotylinae-Xenocelinae’. However, only one mt genome of Languriinae and one mt genome of Xenocelinae were sampled in this study. To better understand the phylogenetic position as well as the relationships within Erotylidae, more mt genomes from Xenocelinae and Languriinae will be needed in the future. Due to the few mt genomes in Cucujoidea, increased taxon sampling is required to resolve the relationships within the superfamily Cucujoidea.

## Figures and Tables

**Table 1:** List of taxonomic groups used for the phylogenetic analyses in this study.

Family	Species	Accession No.	Size (bp)
**Ingroups**			
Cryptophagidae	*Micrambe villosus*	KX087317.1	17,907 bp
Cucujidae	*Cucujus haematodes*	KX087268.1	16,120 bp
Laemophloeidae	*Cryptolestes ferrugineus*	KT182067.1	15,511 bp
Monotomidae	*Monotoma quadricollis*	NC036266.1	16,064 bp
Monotomidae	*Rhizophagus aeneus*	KX087340.1	16,454 bp
Monotomidae	*Rhizophagus dispar*	KX035133.1	13,423 bp
Nitidulidae	*Epuraea guttata*	KX087289.1	16,021 bp
Nitidulidae	*Glischrochilus hortensis*	JX412778.1	10,677 bp
Silvanidae	*Silvanus bidentatus*	KX035145.1	17,220 bp
Silvanidae	*Uleiota* sp.	KX035149.1	14,967 bp
Erotylidae	Languriinae sp.	MG193464.1	16,082 bp
Erotylidae	*Loberonotha olivascens*	JX412784.1	13,039 bp
Erotylidae	*Tritoma metasobrina*	MZ014622	16,502 bp
Erotylidae	*Episcapha opaca*	MZ014623	15,581 bp
Erotylidae	*Neotriplax arisana*	MZ014624	16,478 bp
**Outgroups**			
Curculionidae	*Cyclorhipidion bodoanus*	NC036295.1	15,899 bp
Chrysomelidae	*Calomicrus pinicola*	KX087334.1	15,908 bp

**Table 2:** Annotation of the mitochondrial genome of *T. metasobrina*, *E. opaca*, *N. arisana*, and Languriinae sp.

Gene	Strand	Location		Length (bp)	Anticodon	Codon		Intergenic Sequence (bp)
		Start	End			Start	Stop	
*tRNA^Ile^*	J	1	74/63/67/63	74/63/67/63	CAT			145/61/−3/2
*Trna^Gln^*	N	220/125/65/66	288/193/133/134	69	TTG			3/0/3/3
*tRNA^Met^*	J	292/194/137/138	360/262/205/206	69	GAT			33/18/18/21
*ND2*	J	394/281/224/228	1369/1271/1217/1221	976/991/994/994		ATA/ATA/ATC/ATA	T-tRNA	0
*tRNA^Trp^*	J	1370/1272/1218/1222	1437/1334/1284/1287	68/63/67/66	TCA			0/−8/−1/−1
*tRNA^Cys^*	N	1438/1327/1284/1287	1501/1389/1348/1350	64/63/65/64	GCA			21/0/1/2
*tRNA^Tyr^*	N	1523/1390/1350/1353	1588/1452/1414/1418	66/63/65/66	GTA			−8/0/1/10
*COXI*	J	1581/1453/1416/1429	3120/2987/2946/2950	1540/1535/1531/1522		ATT/TTG/ATT/TTG	T-tRNA	0
*tRNA^Leu(UUR)^*	J	3121/2988/2947/2951	3185/3051/3011/3015	65/64/65/65	TAA			18/0/18/0
*COXII*	J	3204/3052/3030/3016	3876/3738/3695/3700	673/687/666/685		ATA/ATT/ATT/ATT	T-tRNA/TAA/TAA/T-tRNA	0/1/2/0
*tRNA^Lys^*	J	3877/3740/3698/3701	3947/3809/3767/3770	71/70/70/70	CTT			−1/−1/0/0
*tRNA^Asp^*	J	3947/3809/3768/3771	4012/3873/3833/3836	66/65/66/66	GTC			25/18/32/0
*ATP8*	J	4038/3892/3866/3837	4193/4047/4021/3992	156		ATT/ATT/ATT/ATC	TAA/TAA/TAA/TAG	−4/−7/−4/−7
*ATP6*	J	4190/4041/4018/3986	4855/4712/4683/4657	666/672/666/672		ATA/ATG/ATA/ATG	TAA	−1
*COXIII*	J	4855/4712/4683/4657	5642/5498/5469/5445	788/787/787/789		ATG	TA-tRNA/T-tRNA/T-tRNA/TAA	0/0/0/6
*tRNA^Gly^*	J	5643/5499/5470/5452	5708/5561/5534/5517	66/63/65/66	TCC			9/30/12/0
*ND3*	J	5718/5592/5547/5518	6062/5913/5886/5871	345/322/340/354		ATA/ATA/ATA/ATT	TAA/T-tRNA/T-tRNA/TAA	5/0/0/2
*tRNA^Ala^*	J	6068/5914/5887/5874	6132/5980/5955/5939	65/67/69/66	TGC			−1
*tRNA^Arg^*	J	6132/5980/5955/5939	6196/6042/6018/6002	65/63/64/64	TCG			−1
*tRNA^Asn^*	J	6196/6042/6018/6002	6259/6105/6081/6067	64/64/64/66	GTT			0
*tRNA^Ser(AGN)^*	J	6260/6106/6082/6068	6327/6171/6148/6134	68/66/67/67	GCT			1/0/0/0
*tRNA^Glu^*	J	6329/6172/6149/6135	6392/6233/6213/6197	64/62/65/63	TTC			−2/−2/−2/15
*tRNA^Phe^*	N	6391/6232/6212/6213	6455/6293/6277/6277	65/62/66/65	GAA			0
*ND5*	N	6456/6294/6278/6278	8124/7968/7901/7991	1669/1675/1624/1714		ATT/ATA/ATA/GTG	T-tRNA	45/39/90/0
*tRNA^His^*	N	8170/8008/7992/7992	8233/8068/8055/8055	64/61/64/64	GTG			0
*ND4*	N	8234/8069/8056/8056	9568/9401/9388/9388	1335/1333/1333/1333		ATG	TAA/T-tRNA/T-tRNA/T-tRNA	−7
*ND4L*	N	9562/9395/9382/9382	9843/9676/9663/9669	282/282/282/288		ATT/ATA/ATT/ATG	TAA	14/8/8/2
*tRNA^Thr^*	J	9858/9685/9672/6972	9921/9746/9738/9734	64/62/67/63	TGT			0
*tRNA^Pro^*	N	9922/9747/9739/9735	9986/9811/9803/9798	65/65/65/64	TGG			4/1/1/5
*ND6*	J	9991/9813/9805/9804	10,491/10,313/10,311/10,307	501/501/507/504		ATA	TAA	6/11/5/−1
*CytB*	J	10,498/10,325/10,317/10,307	11,629/11,453/11,452/11,446	1132/1129/1136/1140		ATA/ATT/ATA/ATG	T-tRNA/T-tRNA/TA-tRNA/TAA	0
*tRNA^Ser(UCN)^*	J	11,630/11,454/11,453/11,447	11,697/11,520/11,519/11,513	68/67/67/67	TGA			16/16/25/24
*ND1*	N	11,714/11,537/11,545/11,538	12,664/12,487/12,495/12,488	951		TTG/TTG/TTG/ATT	TAG	1
*tRNA^Leu(CUN)^*	N	12,666/12,489/12,497/12,490	12,732/12,550/12,561/12,554	67/62/65/65	TAG			0/29/0/0
*rrnL*	N	12,733/12,580/12,562/12,555	14,009/13,631/13,846/13,933	1365/1052/1285/1379				0/197/0/0
*tRNA^Val^*	N	14,010/13,829/13,847/13,934	14,079/13,893/13,916/14,003	70/65/70/70	TAC			0/0/0/0
*rrnS*	N	14,080/13,894/13,917/14,004	14,850/14,652/14,693/14,777	772/759/777/774				0
A + T rich-region		14,851/14,653/14,694/14,778	16,502/15,581/16,478/16,082	1652/929/1785/1305				0

Note: N and J indicate that the gene was located in the minor (N) and major (J) strand. The ‘/’ indicated that these from left to right were *T. metasobrina*, *E. opaca*, *N. arisana*, and Languriinae sp. Intergenic sequence: positive numbers/negative numbers indicate intergenic/overlapping regions between adjacent genes.

**Table 3:** Base composition and strand bias of these four species.

Species	CG			PCGs			16S rRNA			12S rRNA			CR		
	A + T (%)	AT Skew	GC Skew	A + T (%)	AT Skew	GC Skew	A + T (%)	AT Skew	GC Skew	A + T (%)	AT Skew	GC Skew	A + T (%)	AT Skew	GC Skew
*T. metasobrina*	74.80	0.04	−0.29	72.90	0.04	−0.25	81.70	0.05	−0.31	77.90	0.05	−0.34	75.50	0.04	−0.66
*E. opaca*	78.70	0.03	−0.20	77.79	−0.12	−0.01	80.70	−0.04	0.31	81.03	0.00	0.32	81.59	0.17	−0.27
*N. arisana*	75.39	0.04	−0.23	73.66	−0.15	−0.02	81.48	−0.01	0.34	79.43	0.00	0.31	77.54	0.12	−0.50
* Languriinae sp.	76.46	−0.01	−0.20	74.96	−0.15	−0.02	81.22	0.07	−0.38	78.55	−0.01	−0.31	79.69	−0.02	−0.28

Note: AT skew = (A − T)/ (A + T), GC skew = (G − C)/ (G + C); CG = complete mitogenome; CR = control region, also called the A + T-rich region; PCGs =13 Protein-coding genes. ‘*’ indicate the sequence downloaded from NCBI, others sequenced in this study.

## Data Availability

The data that support the findings of this study are openly available in National Center for Biotechnology Information at https://www.ncbi.nlm.nih.gov/nuccore (accessed on 23 April 2021), reference numbers MZ014622−MZ014624. The PCG123R dataset is available in MendeleyData at http://data.mendeley.com/datasets/v5tsxvz9vf/1 (accessed on 25 May 2021) (DOI: 10.17632/v5tsxvz9vf.2).

## Supplementary Materials

The following is available online at https://www.mdpi.com/article/10.3390/insects12060524/s1, Table S1. Detail of software and kit.
