# Supplementary material for: Three Complete Mitochondrial Genomes of Erotylidae (Coleoptera: Cucujoidea) with Higher Phylogenetic Analysis
Source: Insects. 2021 Jun 5;12(6):524. doi: 10.3390/insects12060524 (PMC8228215; doi:10.3390/insects12060524)
Supplement: Supplementary file 1 [file insects-12-00524-s001.zip › insects-1228726-supplementary.pdf]

Table S1. Detail of software and kit.

**Software/ Kit**	**Resourse**
Geneious 8.0.5	Biomatters, Auckland, New Zealand, http://www.geneious.com/
DNeasy Blood & Tissue kit	QIAGEN, Hilden, Germany
MITOS WebServer	http://mitos.bioinf.uni-leipzig.de/index.py
tRNAscan-SE Search Server v1.21	http://lowelab.ucsc.edu/tRNAscan-SE/
CGView	http://stothard.afns.ualberta.ca/cgview_server
MEGA v 7.313	Penn State University, State College, PA, USA
RNAStructure	https://rna.urmc.rochester.edu/RNAstructure.html
Prism 6.01	GraphPad Software, San Diego, USA
PartitionFinder 2.1.1	www.phylo.org
IQ-TREE v1.6.8	http://www.iqtree.org/
KaKs Calculator 2.0	https://sourceforge.net/projects/kakscalculator2/
ModelFinder	https://www.iqtree.org/ModelFinder/
PhyloSuite	http://phylosuite.jushengwu.com/
PhyloBayes 3	http://www.phylobayes.org
